# Seasonal Variations of C: N: P Stoichiometry and Their Trade-Offs in Different Organs of *Suaeda salsa* in Coastal Wetland of Yellow River Delta, China

**DOI:** 10.1371/journal.pone.0138169

**Published:** 2015-09-22

**Authors:** Fude Liu, Yuhong Liu, Guangmei Wang, Ye Song, Qing Liu, Desheng Li, Peili Mao, Hua Zhang

**Affiliations:** 1 School of Environmental Science and Safety Engineering, Tianjin University of Technology, Tianjin, China; 2 Yantai Institute of Coastal Zone Research, Chinese Academy of Sciences, Yantai, China; 3 College of Biological Sciences and Technology, Beijing Forestry University, Beijing, China; 4 College of Resources and Environment, Qingdao Agricultural University, Qingdao, China; CAS, CHINA

## Abstract

Variations of plant C: N: P stoichiometry could be affected by both some environmental fluctuations and plant physiological processes. However, the trade-off mechanism between them and their influencial factors were not understood completely. In this study, C, N, P contents and their stoichiometry of *S*. *salsa’s* plant organs (leaves, stems, and roots), together with their environmental factors including salinity, pH, soil N and soil P, were examined in the intertidal and supratidal habitats of coastal wetlands during the different sampling times (May, July, September, November). The results showed that both plant organ and sampling times affected C, N, and P and stoichiometry of *S*. *salsa* in the intertidal and supratidal habitats, however, their influencial conditions and mechanisms were different. In the intertidal habitat, the different slopes of C-P and N-P within interspecific organs suggested that plant P, C:P and N:P of *S*. *salsa* were modulated by P concentrations that allocated in the specific organs. However, the slopes of C-N were found to be not significant within interspecific organs, but during the sampling times. These differences of plant N and C:N were related with the physiological demand for N in the specific life history stage. In the supratidal habitat, no significant differences were found in the slopes of C-N, C-P, and N-P within interspecific organs. However, different slopes of C-N among the sampling times also indicated a self-regulation strategy for plant N and C:N of *S*. *salsa* in different ontogenetic stages. In contrast to the intertidal habitat, seasonal variations of P, C:P and N:P ratios within interspecific organs reflected the soil P characteristics in the supratidal habitat. Our results showed that the stoichiometric constraint strategy of plant *S*. *salsa* in this region was strongly correlated with the local soil nutrient conditions.

## Introduction

Carbon (C), nitrogen (N), and phosphorus (P) are three of the most vital elements for plant morphogenesis, and their absorption and allocation prove to be essential for all organisms [1]. The stoichiometry of C, N, and P in tissues can affect some ecological processes such as decomposition, grazing and species composition [2–4], which are connected with the structure, function, and evolution of ecosystems [1, 5]. A relatively constant C: N: P was first concluded from plankton in the ocean [6]. Since then, C: N: P stoichiometry has broadened our understanding of physiological and ecological processes in marine ecosystems [7–9]. Moreover, C: N: P stoichiometry provides an important theoretical, conceptual framework for exploring the relationships between nutrient cycling and biotic feedbacks [10].

Inspired by the findings of C, N, and P ratios in the ocean, the Redfield ratio and related hypotheses were confirmed by the studies in terrestrial and freshwater ecosystems [11–15]. In general, most researchers focused on roles of C, N, and P stoichiometry in the ecological process from individuals to ecosystems [16, 17], studied the cycling of N and P among different tropic levels in food webs [1, 18], and explored the mechanisms of biological feedback and some regulations between environmental conditions and life history strategies of organisms [10, 19]. Although these studies have greatly advanced our knowledge of the elements stoichiometry in aquatic and terrestrial ecosystems, the cycles of mineral elements in organisms are complex. Many experiments have shown that the C: N: P stoichiometry can be affected by abiotic factors, such as temperature, elevation, precipitation, and drought [11, 13, 20, 21], or by human activity [22–25], and biological factors including species compositions, life types, and genotypes [19, 26, 27]. Even for the same species in the same site, plant size, plant ontogeny, plant tissue and sampling date may cause variation of plant stoichiometry [28–31].

Coastal wetlands lie between marine and terrestrial ecosystems, and their plant physiological characteristics of nutrient usage in coastal wetland ecosystems are different from terrestrial and marine environments besides differences in spatial locations. Unfortunately, our understanding of C: N: P stoichiometry affected by plant organs and sampling seasons in the coastal wetlands of the Yellow River Delta was deficient, especially in lack of a mechanism about the trade-offs between biological and environmental constrainted strategies. Although the effects of plant organs and sampling seasons on plant C:N:P stoichiometry were typically reported in the ocean and terrestrial ecosystems [9, 31, 32]. In order to evaluate how plant organ and sampling season affect plant C: N: P stoichiometry, and explore the influencing conditions and mechanisms in coastal wetlands, the species *Suaeda salsa* in the Yellow River Delta was chosen. *S*. *salsa* is an annual halophyte with leaf succulent distributing in the supratidal and intertidal habitats of coastal wetlands of China [33]. Our previous study indicated that *S*. *salsa* in the intertidal habitat prioritized biomass allocation to leaves and reproductive organs, while in the supratidal habitat stems of *S*. *salsa* had greater priority in biomass allocation [34]. These results perhaps indicated different feedback mechanisms of *S*. *salsa* existed in the intertidal and supratidal habitats of coastal wetlands. Therefore, the objectives of this study are (1) to describe the seasonal variations of C, N, P, and C: N: P within different organs of *S*. *salsa* in the Yellow River Delta coastal wetlands, China; (2) to explore the allometric shifts of N and P in reference to C within interspecific organs of *S*. *salsa* during the sampling seasons; (3) to evaluate the effects of plant organs and sampling times on C: N: P stoichiometry of *S*. *salsa*, and to discuss their trade-offs with environmental impacts respectively in the intertidal and supratidal habitats.

## Materials and Method

### Ethics Statement

We carried out this experiment in the Nature Reserve of the Yellow River Delta. All necessary permits from the Nature Reserve management committee of the Yellow River Delta were obtained for the described field study. We confirm that our study had no harm to the environment and the field studies did not involve endangered or protected species.

### Site description

**Fig 1:**
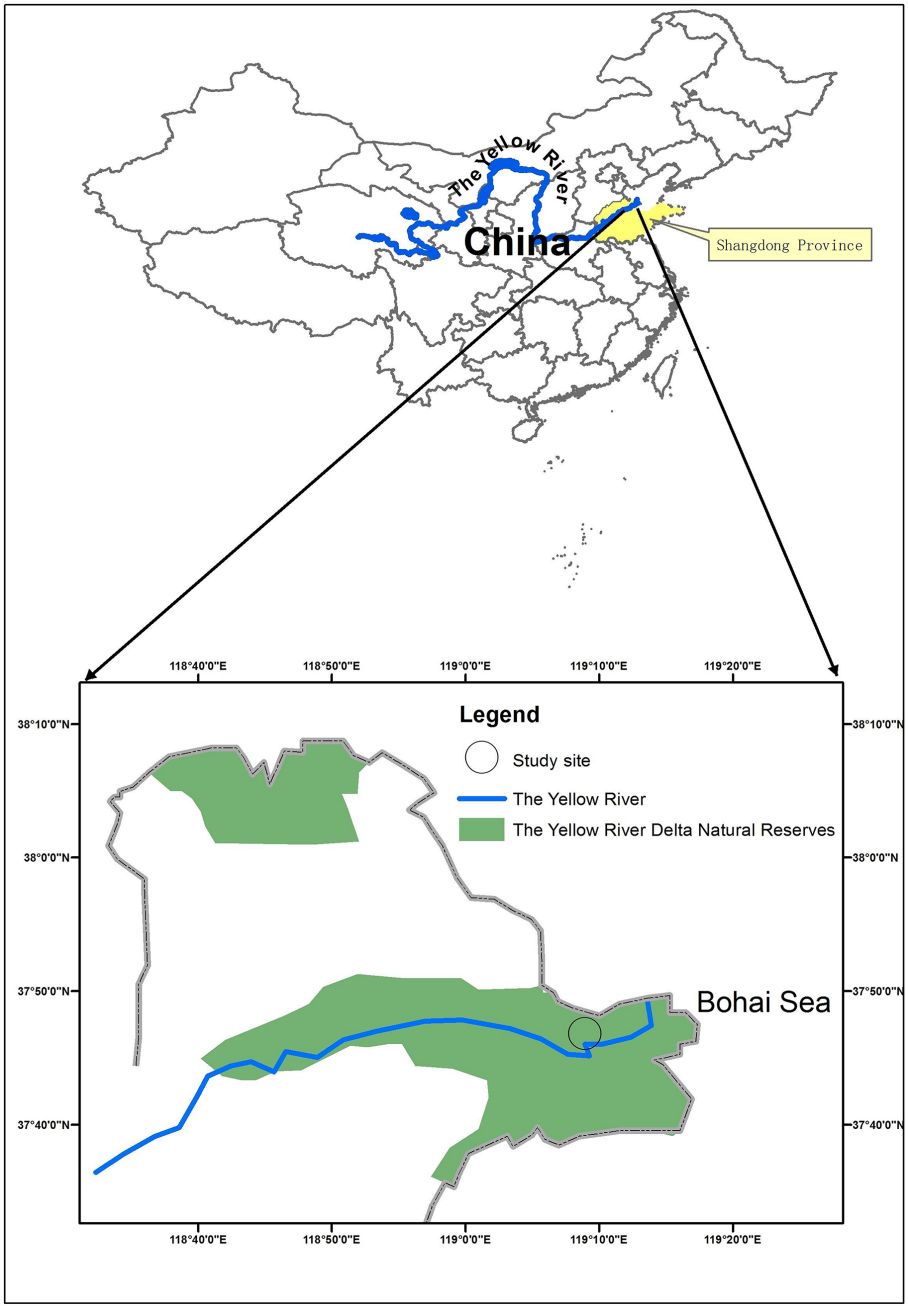
Map of the Yellow River Delta Natural Reserves and the location of our study site.

This study was carried out in the coastal wetlands of the Yellow River Delta located in the Nature Reserve of the Yellow River Delta (37°40′-38°10′N, 118°41′-119°16′E) which covers an area of 1530 km^2^ in Dongying City, Shandong Province, China (Fig 1). In this reserve, the supratidal habitat, the intertidal habitat, and the shallow sea (< 3 m in low-tide) cover 827 km^2^, 382.5 km^2^, and 320.5km^2^, respectively. This region is described by a typical continental monsoon climate. Mean annual temperature, evaporation, and precipitation are 12.3°C, 1926.1 mm, and 542.3 mm, respectively. The frost-free period is 199 days, and the rainy season is from June to August. Intrazonal tidal soil and salty soil are distributed in this region, and the dominant species are *Phragmites australis*, *Tamarix chinensis*, and *S*. *salsa* [35].

### Filed sampling

*S*. *salsa* with high salinity tolerance caused by strong evaporation and tidal inundation is one of the dominant halophytes in the Yellow River Delta [36]. *S*. *salsa* usually germinates in late April, blooms in July, matures in late September, and completely dies in late November [35]. Thus, sampling time (sampling season) was scheduled in May, July, September, and November 2010, and two sampling sites were chosen in the intertidal habitat (37°46′35.6′′N, 119°09′14.6′′E) and supratidal habitat (37°46′61. 3′′′N, 119°09′37. 9′′E). Five quadrats (1 m×1 m) were placed randomly at each site. All plant individuals were collected from each quadrat and separated into leaves, stems, and roots. All the samples were oven dried in the lab at 65°C for 48 h before analyzing their C, N, and P contents. A total of 120 plant samples were collected during the sampling period in 2010. Meanwhile, three soil samples were collected randomly at the depth of 0–10 cm within the same quadrats, and then mixed as one sample. All soil samples were dried and ground into fine powder for laboratory analysis.

### Measurments of plant and soil samples

Total plant C and N concentrations were determined using the elemental analyzer (2400II CHNS/O Elemental Analyzer, Perkin-Elmer, USA), and total plant P concentration was measured by colorimetric method with a Ultraviolet spectrophotometer.

Total soil nitrogen (soil N) was measured using the elemental analyzer (2400II CHNS/O Elemental Analyzer, Perkin-Elmer, USA), and total soil phosphorus (soil P) was measured using the H_2_SO_4_+HClO_4_ digestion.

Soil pH and salinity were measured using the potentiometric method with a soil-water ratio of 1:5 (EC900 Conductivity Analyzer, China).

### Data analysis

All data (S1 Table) were log_10_-transformed to test homogeneity of variance by Levene’s test. One-way analysis of variance (ANOVA) was used to test significant differences in C, N, P, and C: N: P ratios between plant organs and between sampling times, respectively, and then followed by multiple comparison by Tukey’s post-hoc test or the Games-Howell test for heterogeneous variances [37]. A general linear model (GLM) was also applied to evaluate the effects of habitat (intertidal and supratidal habitats), plant organ (leaves, stems, and roots), sampling times (May, July, September, November) and their interactions on the C, N, P stoichiometry of *S*. *salsa*.

A standardized major axis (SMA) regression was used to describe pairwise relationships between elements C, N, and P. Confidence intervals for different regression slopes were calculated by the method of Pitman [38]. The method introduced by Warton and Weber [39] was used to test the heterogeneity of regression slopes and then calculate the common slopes when homogeneity of the slopes occur. Differences in the y-intercept of regression slopes and shifting along the slopes were tested by ANOVA. The calculation of allometric equation parameters was done by using (S) MATR with a Version 2.0 [40]. It should be noted that different roles were explained by slopes and y-intercepts. According to the statistical results, the different regression slopes indicating the physiological constraints for plant C: N: P stoichiometry. When slopes were the same, the different y-intercepts suggesed the environmental influences on plant C: N: P stoichiometry.

## Results

### Soil characteristics in the two habitats

**Fig 2:**
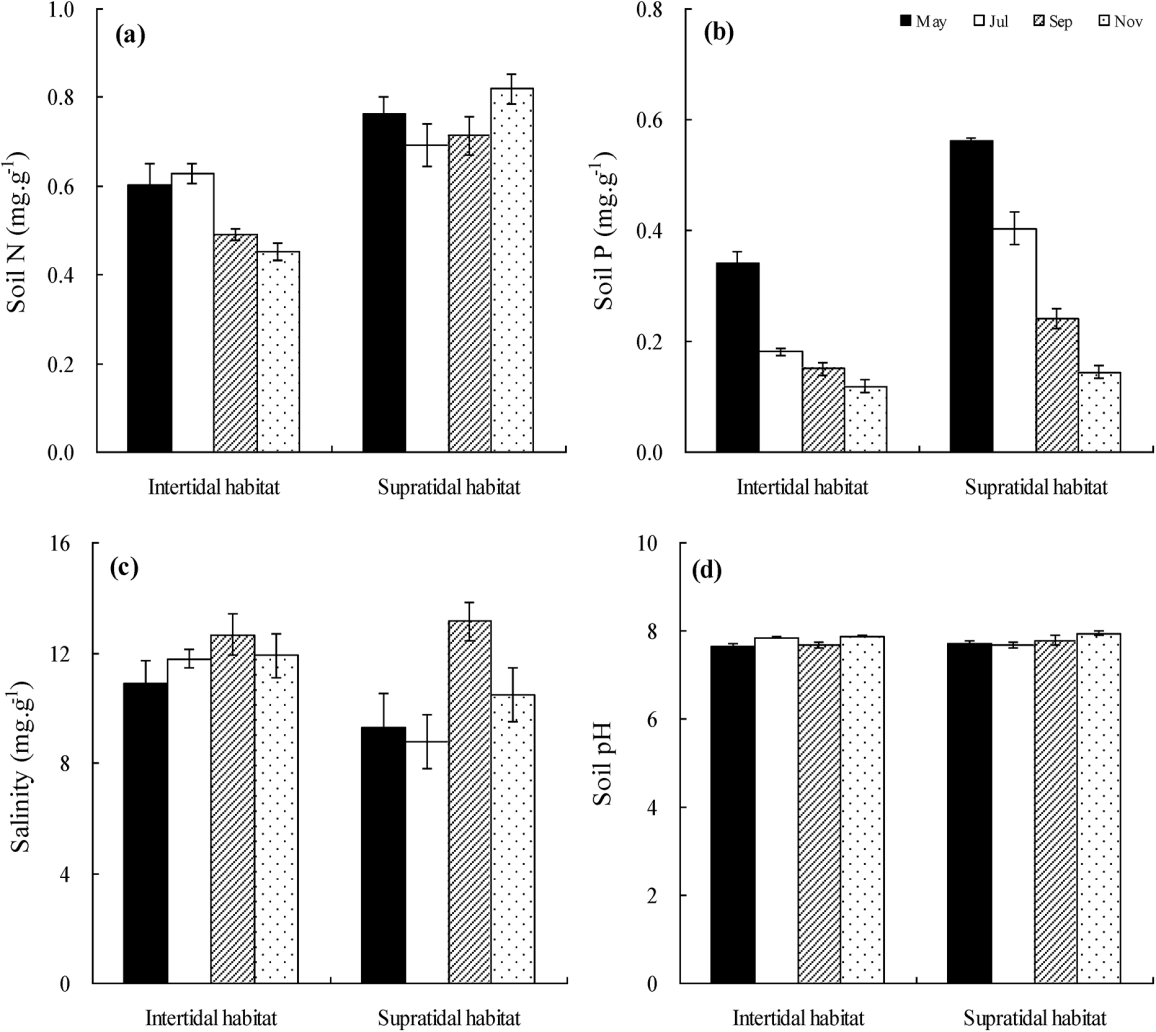
Seasonal variations of soil N, soil P, salinity and pH (Mean ± SE) in the intertidal and supratidal habitats. a. seasonal variations of soil N; b. seasonal variations of soil P; c. seasonal variations of salinity; d. seasonal variations of pH.

In the intertidal zone, N concentrations of soil samples in May and July were higher than in September and November (p<0.05),while no temporal differences were found in the supratidal habitat (p>0.05). By comparision of the two habitats, N concentrations of the intertidal soils were lower than the supratidal soils (p<0.05, Fig 2A). Soil P concentrations decreased obviously with sampling times increasing in the intertidal and supratidal habitats respectively (p<0.05), and were higher in the supratidal habitat than in the intertidal habitat (p<0.05, Fig 2B).

No significant differences were found between sampling times of soil salinity in the intertidal habitat (p>0.05), and salinity of samples collected from September was higher than from other samling times in the supratidal habitat (p<0.05, Fig 2C). No significant differences were found in temperal and spatial distribution of pH (p>0.05, Fig 2D), moreover, the mean average pH in the intertidal and supratidal habitats were both 7.8.

### Variations of C, N, and P concentrations

**Fig 3:**
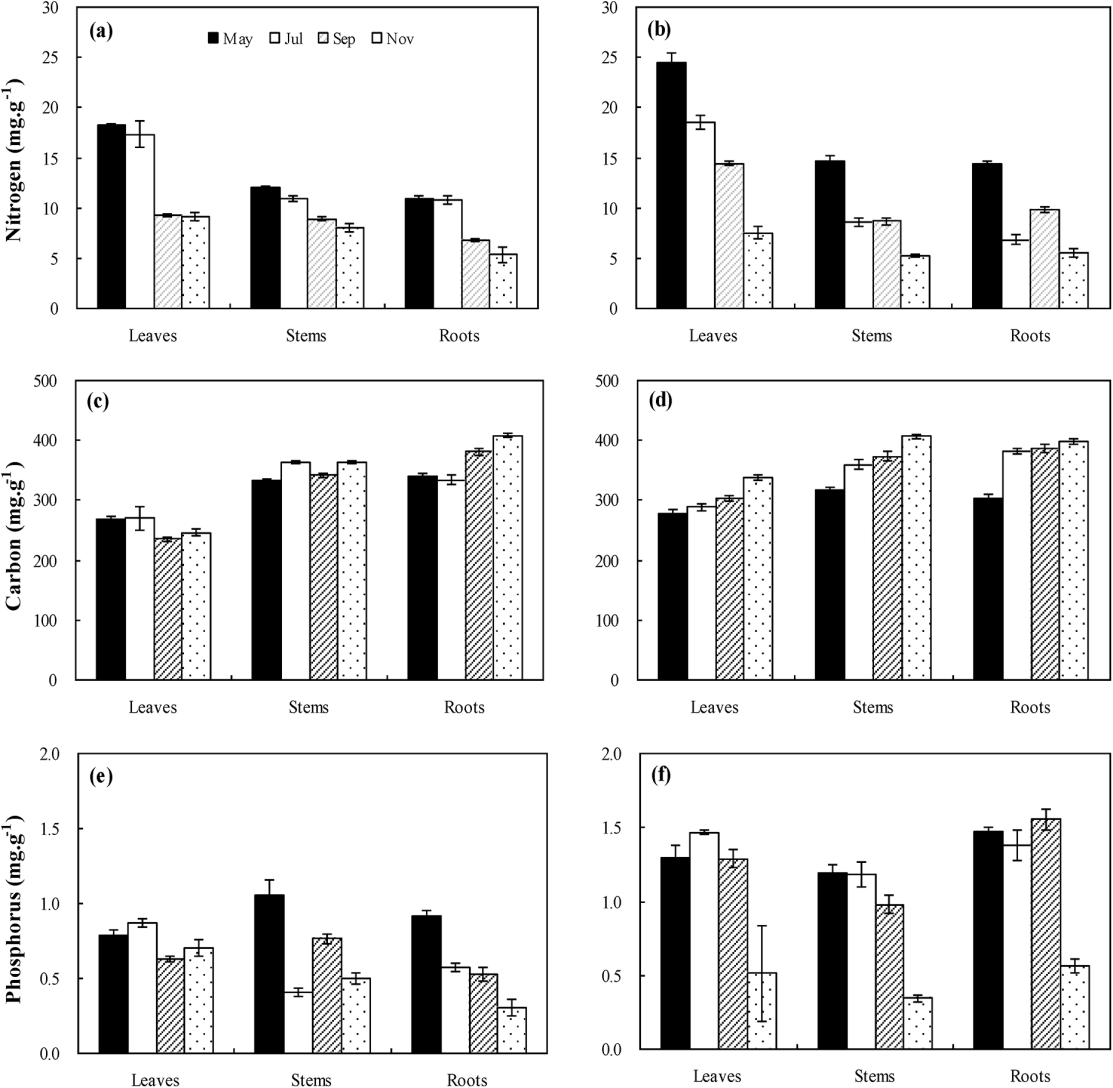
Variations in C, N, and P concentrations (mean ± SE) for specific organs during the life cycle (May, July, September, and November) of *Suaeda salsa* in the intertidal habitat (a, c, and e, respectively) and supratidal habitat (b, d, and f, respectively).

As seen in Fig 3, the large variations of C, N, and P concentrations in the leaf, root, and stem of *S*. *salsa* were observed. In the intertidal habitat, N concentrations varied between 3.59 and 19.95 mg g^-1^, P between 0.19 and 1.43 mg g^-1^, and C between 221.92 and 489.61 mg g^-1^. With growing, the concentration of N decreased in the same organ, and the leaves had higher N concentration than stems and roots. A similar trend existed in the concentrations of P in stems and roots, and no significant variation was found in leaf P concentrations during the different sampling times. For C concentration, no clear trends were found with growing, and average C concentration in the stems and roots was higher than in the leaves (Fig 3A, 3C and 3E). In the supratidal habitat, the concentrations in the root, stem, and leaf varied between 4.59 and 26.14 mg g^-1^ for N, between 0.28 and 1.78 mg g^-1^ for P, and between 264.62 and 420.82 mg g^-1^ for C. N concentration of all three plant organs decreased with growing, and the leaves had higher N concentration than the stems and roots. No obvious differences in P concentration were found among May, July, and September for the same plant organs, and the lowest P concentrations existed in November (P<0.05, Fig 3B, 3D and 3F).

GLM analysis showed that plant organ was the largest contributor (58.19%) for C concentrations, and followed by sampling times (11.03%), whereas the contribution of habitat was only 4.84%. For N concentrations, the largest contributor was sampling times (51.76%), followed by plant organ (27.50%), and no significant effect was found in habitat (0.08%). The largest contributor for P concentrations was also sampling times (41.45%), and followed by the habitat (21.06%). Although the plant organ was found to have significant effect on P concentrations, its contribution was only 2.48% actually (Table 1).

**Table 1:** Results of general linear models for the effects of habitats, sampling time (ST), and plant organ (PO) on C, N, P, and C: N: P stoichiometry during the life cycle of *Suaeda salsa*.

		C		N		P		C: N		C: P		N: P	
Factors	df	MS	SS%	MS	SS%	MS	SS%	MS	SS%	MS	SS%	MS	SS%
Habitat	1	0.026**	4.626	0.003ns	0.078	1.148**	21.064	0.011ns	0.174	0.828**	11.770	1.032**	27.491
Sampling time (ST)	3	0.021**	11.032	0.665**	51.764	0.753**	41.450	0.913**	43.202	1.000**	42.658	0.104**	8.338
Plant organ (PO)	4	0.164**	58.185	0.530**	27.504	0.068**	2.477	1.278**	40.315	0.415**	11.798	0.298**	15.903
Habitat × ST	3	0.009**	4.804	0.101**	7.862	0.187**	10.312	0.134**	6.356	0.206**	8.799	0.257**	20.538
Habitat × PO	2	0.014**	4.982	0.031**	1.635	0.141**	5.156	0.022*	0.678	0.202**	5.729	0.120**	6.393
ST × PO	6	0.004**	4.270	0.021**	3.295	0.042**	4.642	0.018**	1.703	0.059**	5.060	0.044**	7.086
Habitat × ST × PO	6	0.004**	3.737	0.016**	2.543	0.053**	5.853	0.032**	2.997	0.071**	6.084	0.030**	4.768
Residuals	93	0.001	8.363	0.002	5.319	0.005	9.046	0.003	4.574	0.006	8.102	0.004	9.483

Type Ⅲ sums of squares, converted to percentages at each level. Analyses were performed on the entire data from intertidal and supratidal habitats, all data were log_10_-transformed before analysis; d.f. degree of freedom, MS, mean squares, %SS, percentage of sum of squares explained (%); ns indicates not significant (*P* > 0.05)

* indicates statistically significant at the 0.05 significance level (*P<0.05

**P<0.001).

### Variations of C: N: P stoichiometry

**Fig 4:**
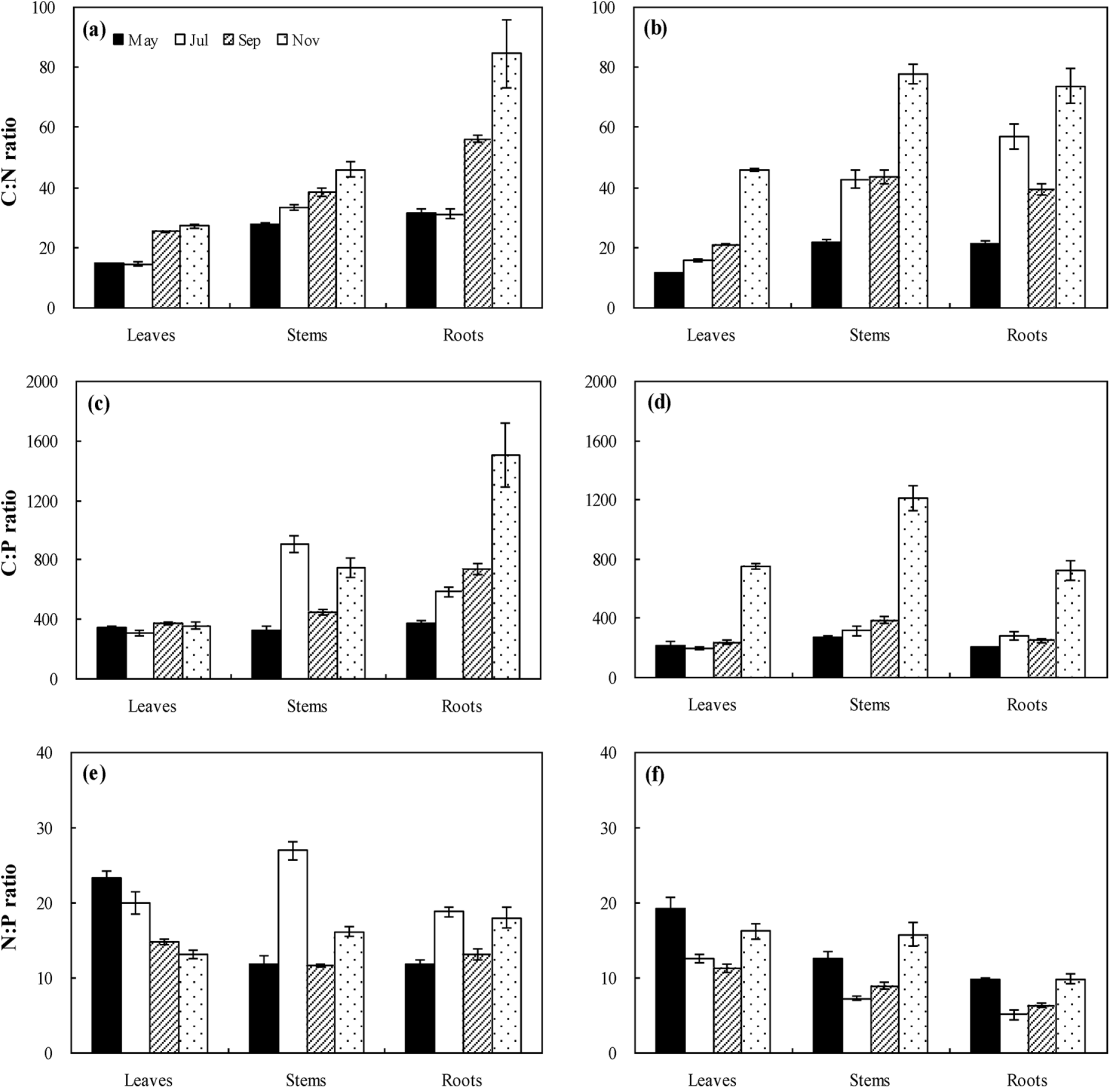
Variations in C: N, C: P, and N: P ratios (mean ± SE) for specific organs during the life cycle (May, July, September, and November) of *Suaeda salsa* in the intertidal habitat (a, c, and e, respectively) and supratidal habitat (b, d, and f, respectively).

C: N, C: P, and N: P of different plant organs in the intertidal habitat ranged from 13.91 to 136.53, 229.03 to 2118.89, and 8.34 to 29.66, respectively. C: N and C: P increased with growth of *S*. *salsa*, and in roots were higher than in leaves and stems (P<0.05, Fig 4A and 4C). N: P of leaves decreased with growth, while no significant trends existed for stems and roots (P>0.05, Fig 4E). In the supratidal habitat, C: N, C: P, and N: P of different plant organs ranged from 10.85 to 88.89, 172.49 to 1451.89, and 6.62 to 24.67, respectively. C: N and C: P increased from May to November, and C: N in the roots and stems was higher than in the leaves (P<0.05, Fig 4B and 4D). Little changes occured in N: P of the different plant organs among different seasons, and the leaves had higher N: P than the roots (P<0.05, Fig 4F).

Based on GLM analysis, sampling times were the largest component (43.20%) contributing to C: N concentrations, followed by plant organs (40.32%), whereas no significant effects of habitats (0.17%) on C: N were found. For C: P variation, 42.66% of them was explained by sampling times, and 11.80% and 11.77% of them explained by plant organ and habitat, respectively, which caused significant effects on C: P,. Habitat is the largest component (27.49%) in explaining N: P variation, and 15.90% and 8.34% of N: P variation were explained by plant organ and sampling season, respectively (Table 1).

### Allometric shifts of C, N, and P relationships

**Fig 5:**
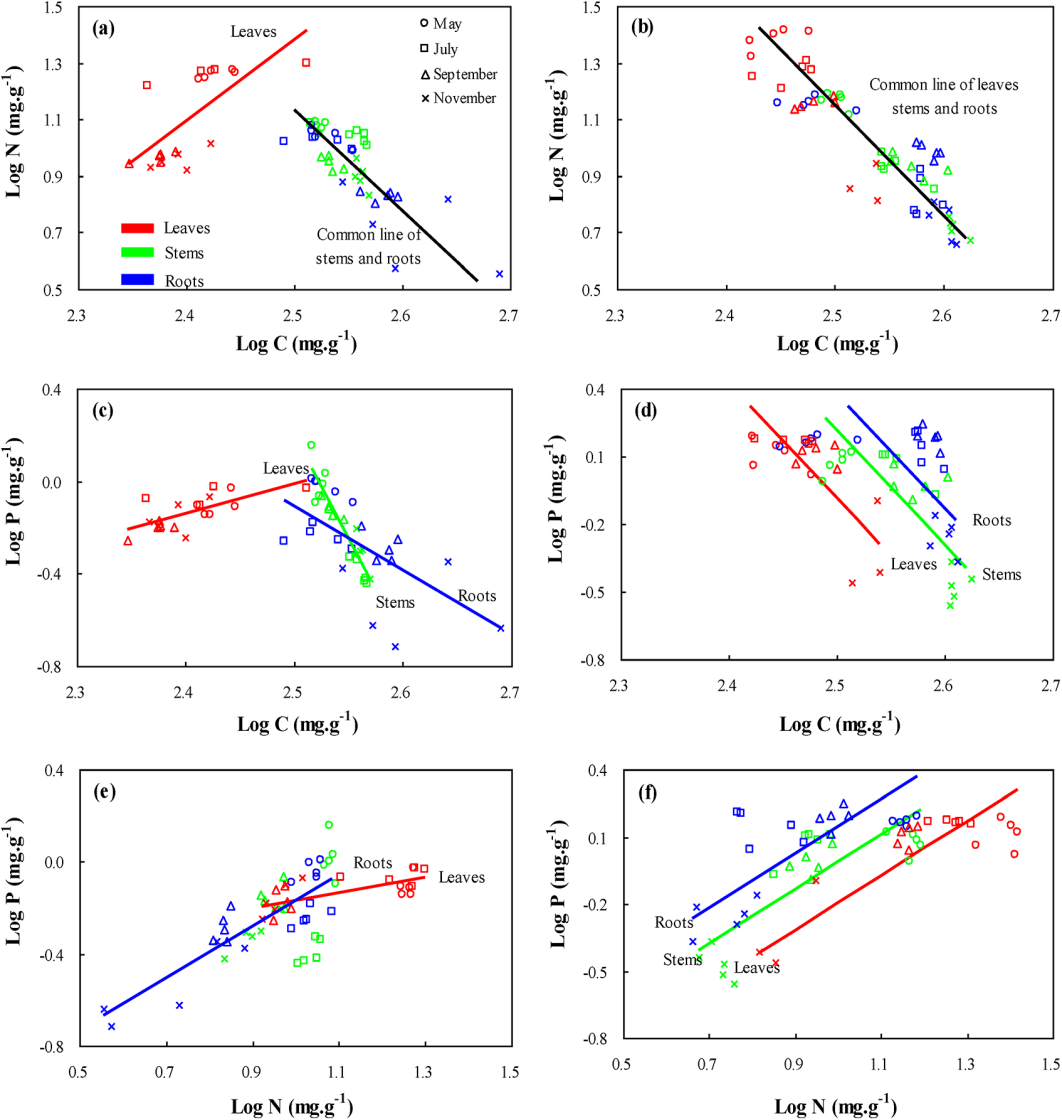
Standardized major axis (SMA) relationships of N and P with respect to C among the different organ types of *Suaeda salsa* during their life cycle in the intertidal habitat (a, c, and e, respectively) and supratidal habitat (b, d, and f, respectively). The SMA regression curves for leaves (red), stems (green), and roots (blue) are respectively shown in figures. However, if there were no significant differences in the slopes and y-intercepts of the regression lines, only the common regression line (black line) is shown. Axes are log_10_ scaled, and results of SMA analyses are presented in Table 2.

For *S*. *salsa* in the intertidal habitat, C concentrations were positively related to N concentrations for the leaves, while negative correlations existed in the stems and roots (Fig 5A). There was an insignificant difference in the slopes of the stems and roots in the space of C versus N and their averaged slope was -3.703 (95% CI = [-3.063, -4.500]). Similar nitrogen allocation patterns existing in the stem and root were indicated by insignificant differences in their y-intercepts (Table 2). For C-P relationships (Fig 5C), the lowest slope was found in the leaves and the highest slope in the stems (Table 2), which indicated that P was prioritized to the leaf compared with the stem and root. In the leaves and roots (Fig 5E), N was positively associated with P, and the steep slope of the leaves indicated higher N: P changes in the leaves compared to the roots (Table 2).

**Table 2:** Results of standardized major axis (SMA) regression analysis for pairwise combinations of carbon (C), nitrogen (N), and phosphorus (P) among the three organs (leaves, stems, and roots) of *Suaeda salsa* in the intertidal and supratidal coastal wetlands of China.

	Leaves				Stems				Roots				Slope homogeneity (*P*)	Shift in elevation (*P*)	Shift along major axis (*P*)
Trait pair X and Y	r^2^	*P*	Slope	Intercept	r^2^	*P*	Slope	Intercept	r^2^	*P*	Slope	Intercept			
Intertidal habitat															
C & N	0.496	**0.001**	4.078	-8.698**a**	0.218	**0.038**	-4.209	11.700**b**	0.701	**<0.001**	-3.333	7.125**b**	0.535	**<0.001**	**<0.001**
C & P	0.472	**0.001**	1.851**a**	-4.586	0.883	**<0.001**	-9.554**b**	24.108	0.418	**0.002**	-4.263**c**	10.640	**0.001**		
N & P	0.525	**<0.001**	0.465**a**	-0.647	0.162	0.079	2.270**b**	-2.451	0.767	**<0.001**	1.279**c**	-1.430	**0.001**		
Supratidal habitat															
C & N	0.607	**<0.001**	-5.151	13.945	0.912	**<0.001**	-3.891	10.897	0.667	**<0.001**	-3.350	9.510	0.145	0.115	**<0.001**
C & P	0.470	**0.002**	-5.506	13.669**a**	0.564	**<0.001**	-5.588	14.219**b**	0.200	**0.048**	-3.823	9.854**c**	0.326	**<0.001**	**<0.001**
N & P	0.654	**<0.001**	1.069	-1.235**a**	0.640	**<0.001**	1.436	-1.433**b**	0.467	**0.001**	1.141	-1.000**c**	0.321	**<0.001**	**0.003**

Statistically significant relationships and differences are shown in bold. Different letters next to individual values in a row indicate statistically significant (*P* < 0.05) pairwise differences in specific organs as determined by Tukey’s multiple comparison tests.

For *S*. *salsa* in the supratidal habitat, C concentrations of the leaves, stems, and roots were negative with N concentrations, and insignificant differences among them produced an averaged slope of -3.924 (95% CI = [-3.473, -4.440]) (Fig 5B). Moreover, a significantly upward shift of leaves data points along the common SMA (Table 2) suggested higher C and N spectra existing in the leaves than in the stems and roots. For C and P, negative correlations existing in leaves, stems, and roots (Fig 5D) with a similar slope of -5.092 (95% CI = [-4.099, -6.285]) and different y-intercepts (Table 2) indicated a higher percentage of P in roots per unit C than in stems and leaves. As can be seen in Fig 5F, N was positively associated with P in the leaves, stems, and roots and their slopes were similar and averaged 1.215 (95% CI = [1.009, 1.465]). The lowest y-intercept in the leaves than in the roots and stems (Table 2) demonstrated the highest N: P in leaves than in the stems and roots at a given N.

## Discussion

Plant N and P concentrations may vary significantly among the different organ types, which can be ascribed to the differences in organs structure and physiology [29]. Our results demonstrated that N concentrations in the leaves of *S*. *salsa* were higher than in the stems and roots, especially in the early sampling times (May and July). As described in previous experiments on woody species, the large amounts of rubisco in photosynthetic organs explained this phenomenon [11, 41]. In contrast to N, no significant differences were found in organ-specific P and that was consistent with the trend reported by Niklas and Cobb in *Eranthis hyemalis* [29]. Since nucleic acid was a major P pool [29, 42] and widely existed in all plant organs, the wide distribution of nucleic acid could decrease the P differences among different organs under the same conditions.

In addition to the variations of N and P levels in organs, leaf N concentration decreased with sampling times increasing from May to November and similar trends existed in the stems and roots. Generally, foliar nutrient concentration decreases with increasing of plant size [19] which was also supported by our results. Interestingly, obvious changes in P concentrations were not found before September but suddenly decreased significantly during the late sampling time (November). Essential nutrients are usually allocated to specific organs for plant survival, which includes nutrient transfer from perennial organs to winterized organs for storage [43, 44], from senescing leaves to reproductive organs for survival [45, 46], and so on. In this study, the decreased N and P concentrations with growing was possibly caused by re-allocation of nutrient to the reproductive organs in late growing seasons. This was confirmed by our measurments of reproductive tissue (flower) biomass of *S*. *salsa* (the concentrations of N and P were 17.01±1.19 and 1.81±0.15 mg g^-1^ in the intertidal habitat, while 18.48±1.51 and 2.77±0.65 mg g^-1^ in the supratidal habitat in November).

Moreover, N and P transfer from one plant organ to another affected seasonal variations of C: N and C: P ratios [29], which was supported by our results that the largest variation in C: N and C: P occurring during the sampling times. In fact, these changes of C: N and C: P were caused by the differences of N and P allocation patterns and the diverse P-rich cellular components within interspecific organs during the sampling times [42]. Thus, the lower C: N in the leaves compared to the stems and roots in the intertidal habitat in our study appears to be the result of a substantial N allocation to rubisco [42, 47]. In the intertidal habitat similar results were found for C: P, however, no clear trends for C: P in the supratidal habitat. The differences of C: P between the two habitats suggested that, the C: N: P stoichiometry of plants could be affected by soil nutrient conditions [19] in addition to the resorption of N and P by plants,.

Growth rate hypothesis (GRH) suggested that N: P ratio was correlated with growth rate negatively [13, 48]. Our data revealed that *S*. *salsa* grown in the intertidal habitat had higher N: P ratio than in the supratidal habitat, which probably resulted from the low growth rate of *S*. *salsa* in the intertidal habitat (data not shown). However, not all studies support the viewpoint of GRH, recent studies reported that the relationship between N: P and growth rate varied obviously under different nutrient conditions [32]. Thus, It was helpful and important for us to understand C: N: P stoichiometric regulation patterns by exploring the soil nutrient conditions in the inter- and supratidal habitats. Our fertilization experiment indicated that the growth of *S*. *salsa* in the supratidal habitat was obviously limited by N (S1 Fig), which was consistent with the conclusion that N was the predominant limiting nutrient in coastal ecosystems [49, 50]. Plant N: P ratios also reflected soil nutrient availability in most ecosystems [11, 51]. Generally, when leaf N: P was less than 14 or more than 16, plant growth was limited more by N or P, while N: P between 14 and 16 meant the equal limitation of N and P on plant growth [52–54]. In the intertidal habitat, the average N:P with 17.84 for leaves of *S*. *salsa* higher than the threshold of 16:1 indicated a potential P-limited condition in this habitat. Moreover, the limitation threshold was proved to be different in interspecific ecosystems and sites [24]. For low soil nutrient concentrations observed during the sampling times (ranged from 0.45 to 0.6 mg g^-1^ for soil N, and 0.12 to 0.34 mg g^-1^ for soil P), we concluded that N and P co-limited condition existed in this habitat.

The slopes or intercepts of the allometric relationships for C-N, C-P and N-P described C: N: P stoichiometric constraint strategies of plants in different habitats [55]. In our study of intertidal habitat, the significant differences in the slopes of C-P and N-P among the roots, stems, and leaves of *S*. *salsa* suggested that C: P and N: P were regulated by different allocation patterns within interspecific organs. The result was consistent with the prediction that the coupling of P to other elements tended to be changeable in geographic scales [19].However, the slopes of C-N did not show significant differences for the leaves, stems, and roots, but among the sampling times (S2 Fig, S2 Table). This possibly related to the physiological demand for N in the specific life history stage. In the supratidal habitat, the slopes of C-N, C-P, and N-P relationships were not significantly different among the different organs of *S*. *salsa*, and this proved the similarities of nutrient allocation in the different organs. Notably, the significantly different slopes of C-N relationships among the sampling times (S2 Fig, S2 Table), indicated the differences of the physiological demand for N in the specific life history stage. Some studies showed that the various intercepts with a similar slope meant that the nutrient concentrations in species (or organ types) were affected directly by environmental factors [55, 56]. In this study, the y-intercepts of C-P and N-P changed significantly from leaves to stems and roots of *S*. *salsa*, and richer soil P relative to soil N decreased with sampling seasons increasing. These results indicated that C: P and N: P ratios produced a response to seasonal variations of soil P in the supratidal habitat.

As we all known, variations of plant C: N: P stoichiometry in different sites can mirror specific geographic environment, and/or it may reflect the physiological requirement of plant [19, 31]. Our study also suggests that variation of C: N: P stoichiometry may be caused by the different nutrient allocation patterns within interspecific organs or physiological demands in different sampling times. Alternatively, it may reflect the environmental fluctuations in specific sampling seasons. Additionally, our results indicate that those trade-offs between biological and environentmal constraints are strongly correlated with the local soil nutrient conditions. In summary, the findings will provide some novel insights into the roles of C:N:P stoichiometric constraint strategy for plant, and improve our understanding of ecological processes in the coastal wetlands of Yellow River Delta.

## Supporting Information

S1 Fig Mean ± SE of biomass, N, and P concentrations in specific organs of *Suaeda salsa* under the field nutrients addition experiment. **a**. variations in biomass for specific organs; **b**. variations in N concentration for specific organs; **c**. variations in P concentration for specific organs. Control represents soil with no N and P addition; +N represents soil with N addition; +P represents soil with P addition; and N+P represents soil with N and P addition.
(TIF)

S2 Fig Standardized major axis (SMA) relationships of N and P with respect to C across the different sampling times in the intertidal habitat (a, c, and e, respectively) and supratidal habitat (b, d, and f, respectively). The SMA regression curves for sampling season in May, July, September and November are respectively shown in figures. However, if there were no significant differences in the slopes and y-intercepts of the regression lines, only the common regression line is shown. Axes are log_10_ scaled, and results of SMA analyses are presented in S2 Table.
(TIF)

S1 Table Plant and soil data are used in this study. (XLS)

S2 Table Results of standardized major axis (SMA) regression analysis for pairwise combinations of carbon (C), nitrogen (N), and phosphorus (P) among the different sampling times (May, July, September and November) for *Suaeda salsa* in the intertidal and supratidal coastal wetlands of China. Statistically significant relationships and differences are shown in bold. Different letters next to individual values in a column indicate statistically significant (*P* < 0.05) pairwise differences in different sampling times as determined by Tukey’s multiple comparison tests.
(DOC)
