# Supplementary figures and images for: Seasonal Variations of C: N: P Stoichiometry and Their Trade-Offs in Different Organs of Suaeda salsa in Coastal Wetland of Yellow River Delta, China
Source: PLoS One. 2015 Sep 22;10(9):e0138169. doi: 10.1371/journal.pone.0138169 (PMC4578878; doi:10.1371/journal.pone.0138169)

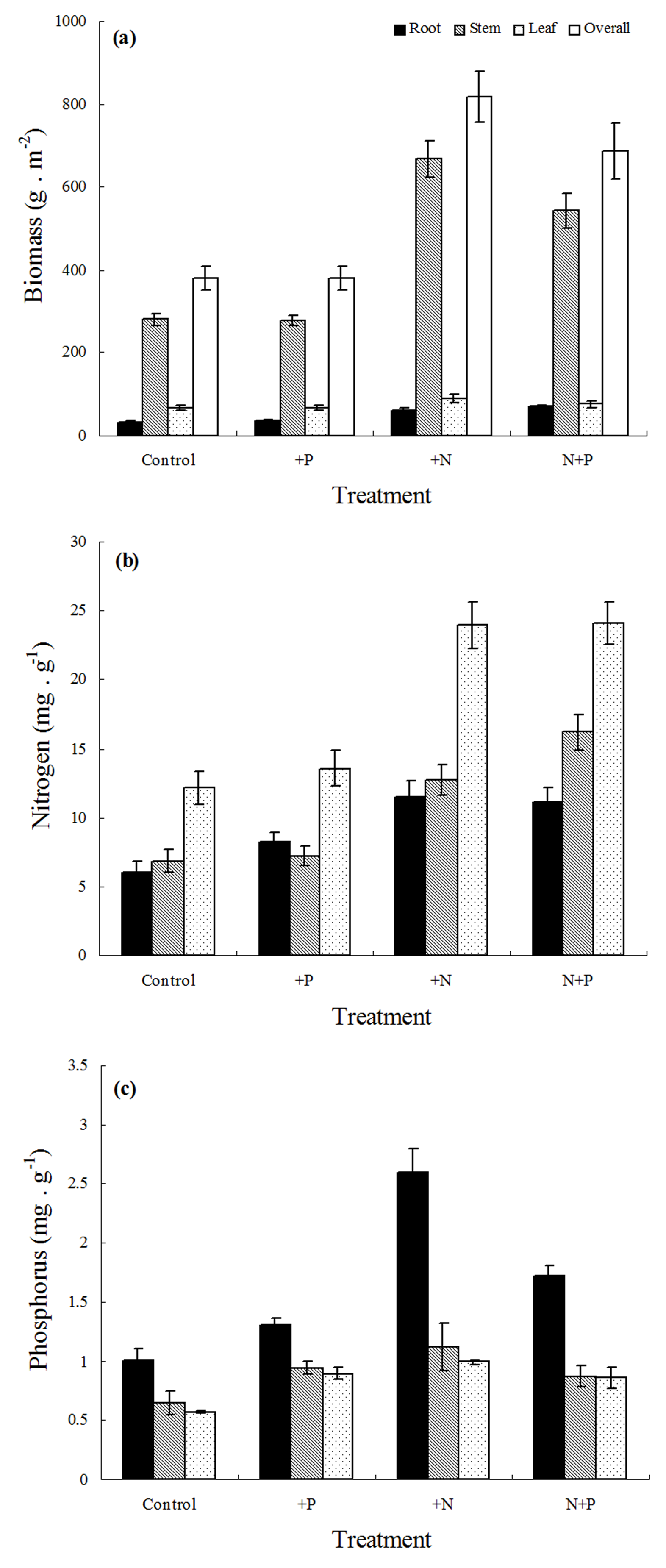

Supplement: S1 Fig — a. variations in biomass for specific organs; b. variations in N concentration for specific organs; c. variations in P concentration for specific organs. Control represents soil with no N and P addition; +N represents soil with N addition; +P represents soil with P addition; and N+P represents soil with N and P addition. (TIF) [file pone.0138169.s001.tif]

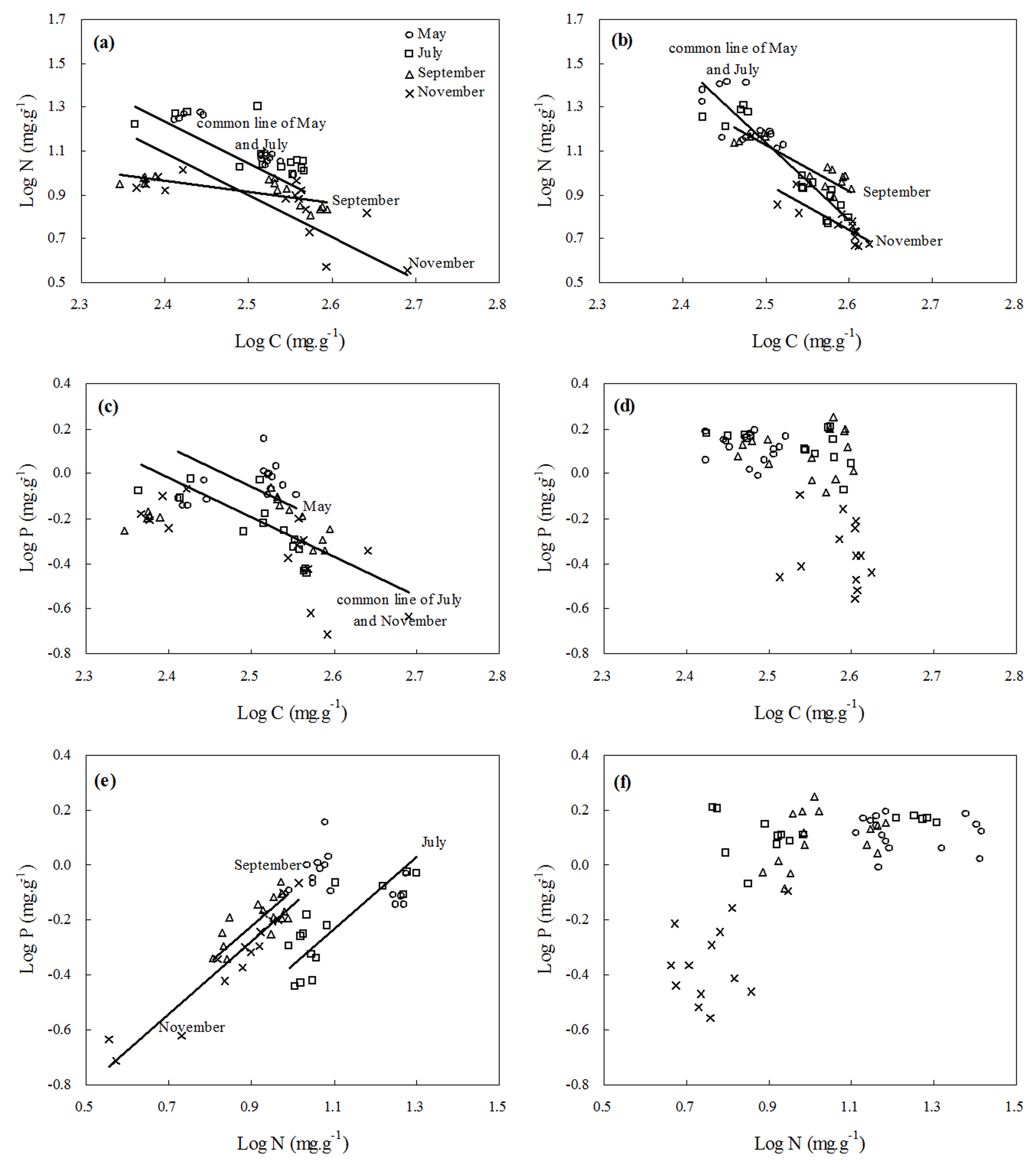

Supplement: S2 Fig — The SMA regression curves for sampling season in May, July, September and November are respectively shown in figures. However, if there were no significant differences in the slopes and y-intercepts of the regression lines, only the common regression line is shown. Axes are log10 scaled, and results of SMA analyses are presented in S2 Table. (TIF) [file pone.0138169.s002.tif]
