# Supplementary material for: Seasonal Variations of C: N: P Stoichiometry and Their Trade-Offs in Different Organs of Suaeda salsa in Coastal Wetland of Yellow River Delta, China
Source: PLoS One. 2015 Sep 22;10(9):e0138169. doi: 10.1371/journal.pone.0138169 (PMC4578878; doi:10.1371/journal.pone.0138169)
Supplement: S2 Table — Statistically significant relationships and differences are shown in bold. Different letters next to individual values in a column indicate statistically significant (P < 0.05) pairwise differences in different sampling times as determined by Tukey’s multiple comparison tests. (DOC) [file pone.0138169.s004.doc]

**S2 Table. Results of standardized major axis (SMA) regression analysis for pairwise combinations of carbon (C), nitrogen (N), and phosphorus (P) among the different sampling times (May, July, September and November) for *Suaeda salsa* in the intertidal and supratidal coastal wetlands of China.**

| **Groups of sampling times** | **C & N** | | | | **C & P** | | | | **N & P** | | | |
| --- | --- | --- | --- | --- | --- | --- | --- | --- | --- | --- | --- | --- |
|  | r^2^ | *p* | Slope | Intercept | r^2^ | *p* | Slope | Intercept | r^2^ | *p* | Slope | Intercept |
| **Intertidal habitat** | | | | | | | | | | | | |
| May | 0.922 | **<0.001** | -2.046**a** | 6.227 | 0.278 | **0.043** | 1.592 | -4.014**a** | 0.241 | 0.063 | -0.778 | 0.832**ad** |
| July | 0.563 | **0.002** | -1.740**a** | 5.467 | 0.638 | **0.001** | -2.225 | 5.340**b** | 0.648 | **<0.001** | 1.346 | -1.714**b** |
| September | 0.530 | **0.002** | -0.696**b** | 2.654 | 0.034 | 0.511 | -0.885 | 2.008**b** | 0.539 | **0.002** | 1.271 | -1.366**ac** |
| November | 0.492 | **0.004** | -1.355**c** | 4.272 | 0.536 | **0.002** | -1.901 | 4.456**b** | 0.904 | **<0.001** | 1.403 | -1.537**d** |
| Slope homogeneity (*P*) | **0.003** | | | | 0.057 (common slope = -1.748) | | | | 0.182 (common slope = 1.320) | | | |
| Shift in elevation (*P*) |  | | | | **<0.001** | | | | **<0.001** | | | |
| Shift along major axis (*P*) |  | | | | **<0.001** | | | | **<0.001** | | | |
| **Supratidal habitat** | | | | | | | | | | | | |
| May | 0.456 | **0.006** | -3.647**a** | 10.263 | 0.018 | 0.637 | -1.986 | 5.031 | 0.017 | 0.644 | -0.545 | 0.789 |
| July | 0.870 | **<0.001** | -3.525**a** | 3.933 | 0.252 | 0.056 | -1.279 | 3.361 | 0.119 | 0.208 | 0.363 | -0.243 |
| September | 0.787 | **<0.001** | -2.009**b** | 6.145 | 0.000 | 0.976 | -1.938 | 5.031 | 0.117 | 0.212 | 0.965 | -0.899 |
| November | 0.688 | **<0.001** | -2.362**c** | 6.874 | 0.028 | 0.586 | -4.147 | 10.376 | 0.140 | 0.208 | 1.756 | -1.690 |
| Slope homogeneity (*P*) | **0.013** | | | |  | | | |  | | | |
| Shift in elevation (*P*) |  | | | |  | | | |  | | | |
| Shift along major axis (*P*) |  | | | |  | | | |  | | | |

Statistically significant relationships and differences are shown in bold. Different letters next to individual values in a column indicate statistically significant (*P* < 0.05) pairwise differences in different sampling times as determined by Tukey’s multiple comparison tests.
